# Prognostic Role of EMR1 in Lymph Nodes and Primary Tumors of Colon Cancer Patients

**DOI:** 10.1002/adbi.202500645

**Published:** 2026-08-14

**Authors:** Tamer Roshdy, Hagar Eltorky, Alaa Mohamed, Faten Zahran, Adel Guirgis, Lina Olsson, Gudrun Lindmark, Marie‐Louise Hammarström, Sten Hammarström, Basel Sitohy

**Affiliations:** ^1^ Department of Clinical Microbiology Umeå University Umeå Sweden; ^2^ Department of Diagnostics and Intervention, Oncology Umeå University Umeå Sweden; ^3^ Department of Molecular Biology, Genetic Engineering, and Biotechnology Research Institute University of Sadat City Sadat City Menoufia Egypt; ^4^ Department of Biochemistry Faculty of Science Zagazig University Zagazig Egypt; ^5^ Institution of Clinical Sciences Lund University Lund Sweden

**Keywords:** CEA, CXCL16, CXCL17, EMR1, eosinophilic biomarker, LGR6, regional lymph nodes

## Abstract

The purpose was to investigate whether the eosinophil biomarker EMR1, ectopically expressed in colon cancer tumor cells, could be used to identify patients at risk for relapse after curative surgery. EMR1 mRNA levels were determined in primary tumors (*n* = 66) and lymph nodes (LNs; *n* = 377) from 121 colon cancer patients. Controls were resection margins of colon cancer tumors and LNs from patients with inflammatory bowel disease. Primary read‐out was recurrency evaluated as disease‐free survival time in the Kaplan–Meier survival model and hazard ratio calculation by uni‐ and multivariate Cox regression. High EMR1 levels in the regional LNs were associated with poor prognosis [hazard ratio 3.1 (1.2–8.0) at 5‐year follow‐up, *p* = 0.02]. Patients with a very poor prognosis were identified by combining EMR1 with CXCL17 analysis of LNs [hazard ratio 4.6 (1.1–19.7), *p* = 0.04]. High EMR1 levels in the primary tumor were associated with a good prognosis [hazard ratio 0.1 (0.02–0.9), *p* = 0.04]. Multivariate analysis strengthened the result for LNs but weakened the result for the primary tumor. These contradictory prognostic results probably relate to the prominent eosinophilia in the primary tumor, essentially lacking in LNs, where ectopically expressed EMR1 in tumor cells dominates. Thus, EMR1 expressed in tumor cells probably promotes tumor growth.

## Graphical abstract

This study evaluated the prognostic value of the eosinophil biomarker EMR1 in 121 colon cancer patients. While high EMR1 levels in primary tumors correlated with improved survival, elevated levels in regional lymph nodes predicted poor outcomes. Combining EMR1 with CXCL17 further identified high‐risk patients. These findings suggest that EMR1's clinical impact varies significantly between primary tumors and lymph nodes.

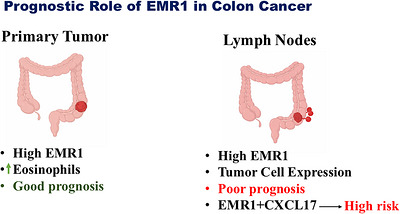

## 1 Introduction

Colorectal cancer (CRC) ranks among the leading causes of cancer‐related mortality and is the third most prevalent form of malignancy worldwide, with a growing frequency [1]. The primary treatment strategy for CRC is still surgery, which is often combined with other treatments such as radiotherapy, chemotherapy, anti‐angiogenic therapy, and immunotherapy [2, 3, 4, 5]. Approximately 25% of CRC patients undergoing curative resection experience recurrence, resulting in significant mortality rates [6, 7]. Identifying high‐risk patients is crucial in order to provide them with the best possible postoperative treatment options and follow‐up care.

Tumor progression in colon cancer involves tumor cells migrating from the primary intestinal site to form colonies in the regional lymph nodes (LNs) and finally in peripheral organs like the liver and lung. It will depend on the properties of the tumor cells themselves and the microenvironment, including growth‐promoting activated fibroblasts and the protective forces of innate and adaptive immunity. Studies exhibited that CD8^+^T cells contribute significantly to adaptive immune defense, while eosinophils constitute an important factor of the innate defense against cancer [8, 9]. Eosinophils and CD8^+^T cells both have cytotoxic effector functions able to affect tumor cell viability upon close contact. High eosinophil numbers in primary malignant tumor stroma were positively correlated to the patient's 5‐year CRC‐specific and overall survival, as demonstrated by Prizment and coworkers' study in which a specific monoclonal antibody was used to quantify eosinophils [10]. That study confirms previous results with the classical staining method to score for eosinophils [11, 12, 13]. Thus, eosinophils appear to have a protective role in the primary tumor, and the amount was inversely correlated to tumor stage [14]. Metastatic LNs of CRC patients generally had only low levels of eosinophils (<40 eosinophils/mm^2^), and whether they exert a protective role or not is unclear [14].

In an earlier study, we used the biomarker epidermal growth factor‐like module‐containing mucin‐like hormone receptor‐like 1 (EMR1/ADGRE1) to detect eosinophils in colon cancer cell lines and in a limited number of primary tumors and LNs of colon cancer patients using immunohistochemistry and mRNA analysis [15]. Unexpectedly, we found that EMR1 protein was ectopically expressed in the tumor cells themselves, while normal colon epithelium did not express EMR1. Immuno‐morphometric analysis, according to Weibel, revealed EMR1‐positive staining in 20% of malignant tumor cells within primary colon cancer tumors, with a large variation between patients. In that study, we also found that EMR1 mRNA was highly expressed in the human monocyte cell line U937, confirming results from the Human Protein Atlas and Hamann et al. [16, 17], which furthermore demonstrated low levels of EMR1 in monocytes and basophils. EMR1 belongs to the group‐II adhesion G protein‐coupled receptor (aGPCR) subfamily, characterized by epidermal growth factor‐like seven‐transmembrane receptors [18]. The ligand for EMR1 is not known.

Ectopic expression of the biomarkers CXCL17, GPR35, FOXQ1, and THBS2 has been demonstrated in 10% to 30% of primary colon cancer tumor cells. Most interestingly, elevated levels of expression of these biomarkers in patients' LNs were in all cases associated with poor prognosis [19, 20, 21, 22]. Since EMR1 also shows ectopic expression in tumor cells, we asked the question whether EMR1 could be used to predict outcome. Therefore, we constructed a real‐time quantitative reverse transcriptase‐polymerase chain reaction (qRT‐PCR) test with RNA copy standard detecting all five splice variants of EMR1. This assay was utilized to analyze primary tumors and LNs from a larger clinical material of colon cancer patients. Primary read‐out was recurrence after curative surgery.

## 2 Results

### 2.1 EMR1 mRNA Expression Profile in Primary Colon Cancer Tumor and Normal Colon Tissue

**FIGURE 1:**
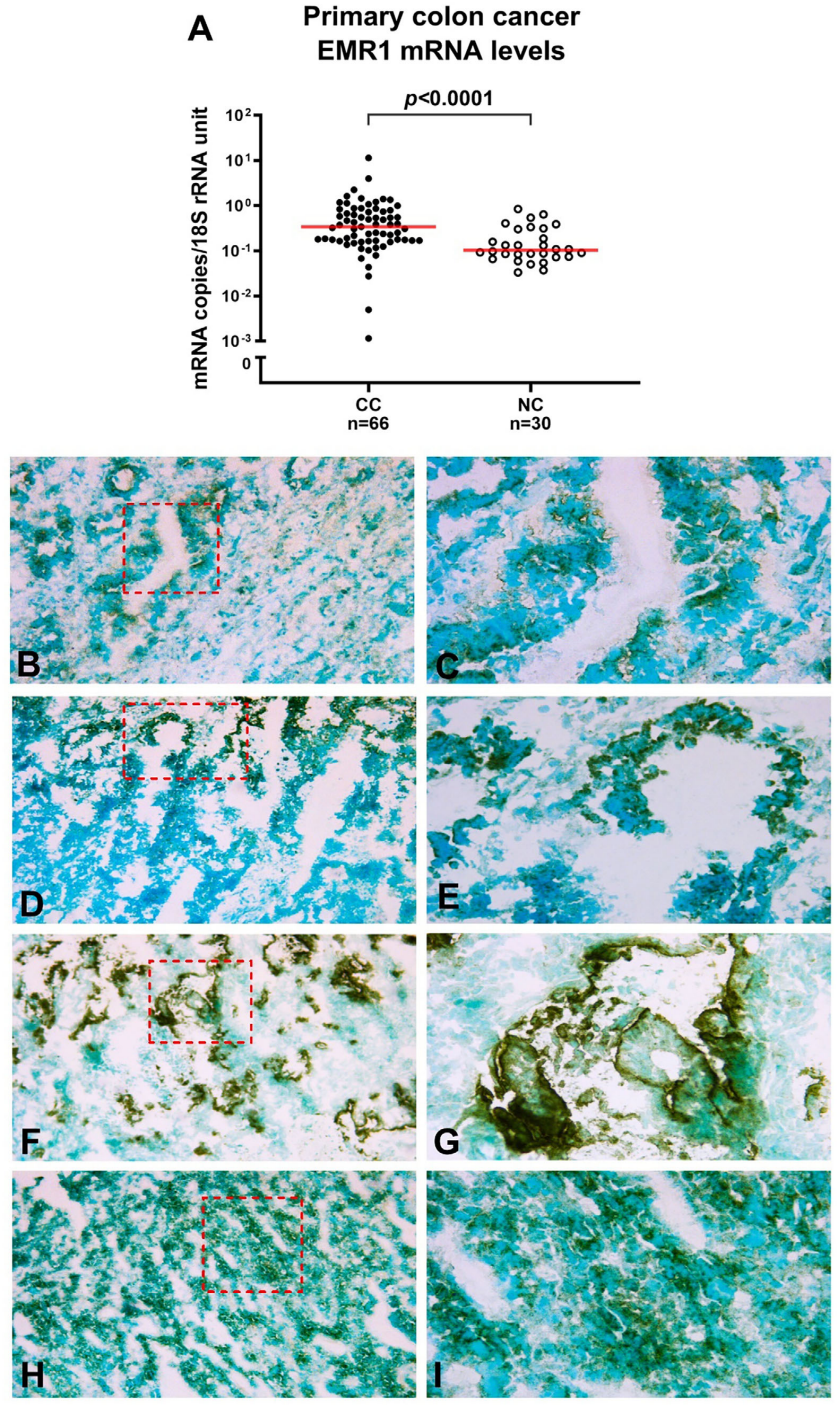
EMR1 mRNA and protein expression in colon cancer primary tumor. (A) Levels of EMR1 mRNA in the primary tumor of colon cancer patients (CC, filled dots) compared to normal colon tissue (NC, open dots). Each dot represents an individual sample, with one sample per patient. mRNA levels are expressed as copies per 18S rRNA unit, and red horizontal bars indicate the median values. Sample sizes are given as *n*‐values and were tissue from 66 colon cancer patients (CC) and 30 control patients (NC). The *p*‐value from two‐sided Mann–Whitney U test is given in the graph. A *p‐*value ≤ 0.05 was considered statistically significant. (B–I) Anti‐EMR1 immunoperoxidase staining of frozen sections of four primary colon cancer tumors with different EMR1 mRNA levels. The EMR1 mRNA level was 0.34 copies per 18S rRNA unit in (B, C), 0.16 copies per 18S rRNA unit in (D, E), 3.96 copies per 18S rRNA unit in (F, G), and 11.32 copies per 18S rRNA unit in (H, I). (B, D, F, H) Original magnification, 100×. (C, E, G, I) Areas indicated by a box with hatched red line in micrographs (B, D, F, H), respectively, shown at an original magnification of 400×.

Primary colon cancer tumors exhibited significantly higher EMR1 mRNA expression levels than normal colon tissue (*p* < 0.0001). The difference was three‐fold (i.e., 0.3 vs. 0.1 mRNA copies/18S rRNA unit) (Figure 1A).

Primary colon cancer tissue for protein analysis by immunohistochemistry was available for 10 of the tumors. Figure 1B–I show staining of four of these tumors. The left part of the panel shows the original magnification (100×) and the right part 400× magnification of the indicated area. As can be seen, anti‐EMR1 antibodies stain tumor cells in the tissue, and the strongest staining is found in tissue from the patients expressing the highest EMR1 mRNA levels (Figure 1F,H). However, due to the limited sample size, statistical analysis is not meaningful.

### 2.2 EMR1 mRNA Expression in Regional Lymph Nodes of Colon Cancer Patients

**FIGURE 2:**
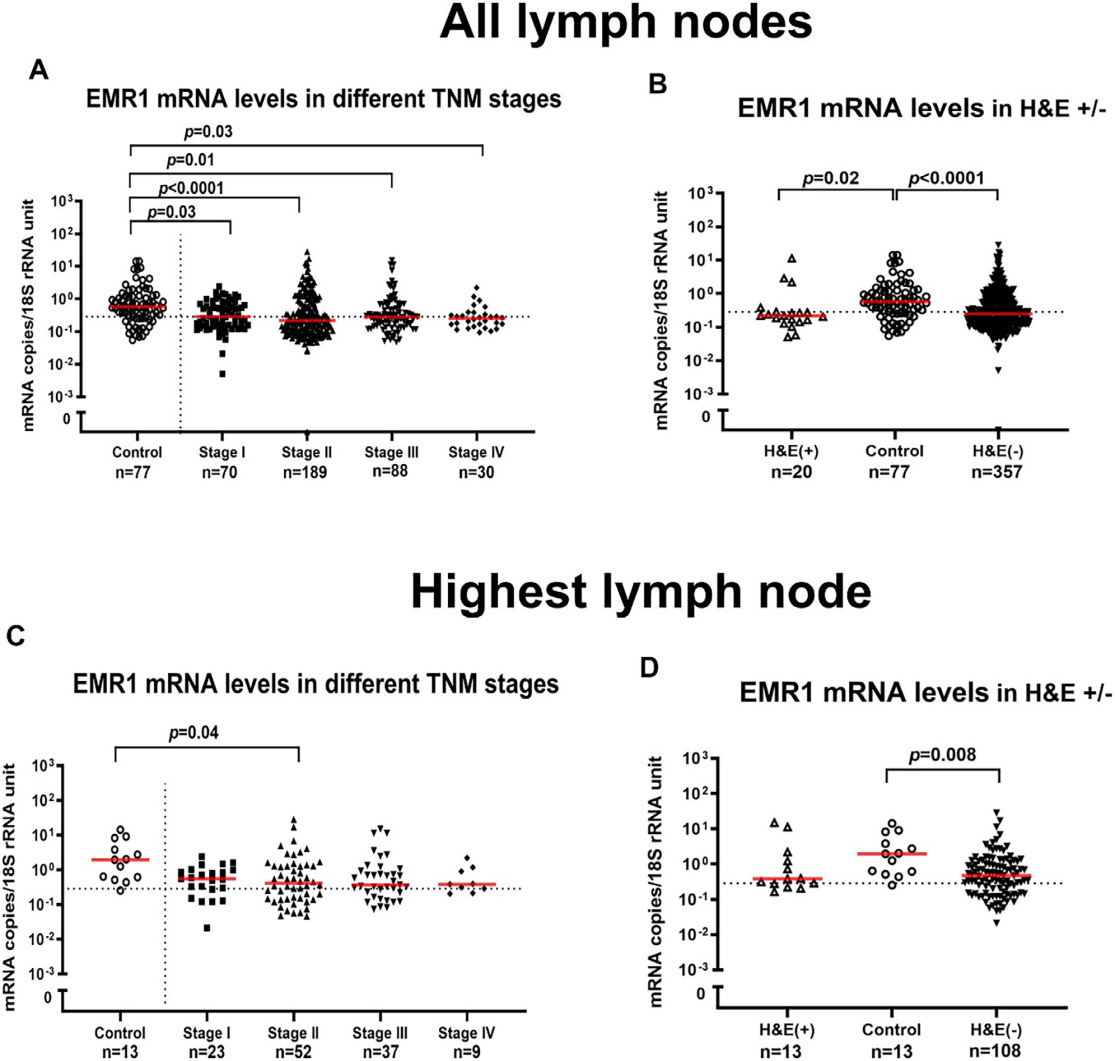
Levels of EMR1 mRNA in lymph nodes of colon cancer patients (*n* = 377) and non‐cancerous disease patients (Control, *n* = 77). (A, C) Lymph nodes of colon cancer patients grouped according to TNM stage [Stage I, Stage II, Stage III, Stage IV] and (B, D) whether they were metastatic [H&E(+)] or not [H&E(‐)]. (A, B) display the result from all lymph nodes analyzed. (C, D) display the results when each patient is represented by the lymph node with the highest EMR1 mRNA level. Each lymph node is represented by an individual symbol in the graph (open circle, lymph node of control patient; filled square, lymph node of Stage I cancer patient; filled triangle pointing upward, lymph node of Stage II cancer patient; filled triangle pointing downward, lymph node of Stage III cancer patient in (A) and (C) and H&E(‐) lymph node in (B) and (D); diamond, lymph node of stage IV cancer patient; open triangle, H&E(+) lymph node). *n* = number of samples. mRNA levels are given as mRNA copies per 18S rRNA unit, and red horizontal bars indicate the median values. Hatched horizontal line represents the 33^rd^ percentile determined from the values of the highest lymph node of colon cancer patients, i.e., the adapted cut‐off for allocation of patients to EMR1(+) and EMR1(‐) groups in survival analysis. Statistical significance was assessed using two‐sided Kruskal–Wallis non‐parametric ANOVA, followed by post hoc Dunn's test for multiple comparisons. Comparisons with a *p*‐value ≤ 0.05 were considered statistically significant, and the *p*‐value for all comparisons with a *p*‐value ≤ 0.05 are given in the graphs.

A cohort of 121 colon cancer patients (TNM stages I–IV) donated 377 LNs, and 13 control patients donated 77 LNs. All LN samples underwent EMR1 mRNA expression analysis. EMR1 mRNA expression levels in LNs were almost the same independent of which TNM stage the patient was classified as belonging to (Figure 2A). Median EMR1 mRNA expression levels, as copies/18S rRNA unit, were 0.3 for stage I, 0.2 for stage II, 0.3 for stage III, and 0.3 for stage IV, respectively. LNs from control patients exhibited significantly higher median EMR1 mRNA levels (0.6 copies/18S rRNA unit) compared to colon cancer patients across all TNM stages (*p* = 0.03, *p* < 0.0001, *p* = 0.01, and *p* = 0.03, respectively).

The LNs were then related to metastasis detection by H&E staining (Figure 2B). Twenty LNs were metastatic [H&E(+)] and 357 LNs were non‐metastatic [H&E(‐)]. The median EMR1 mRNA expression level was 1.5‐fold higher in H&E(‐) than in H&E(+) LNs. This difference was statistically insignificant. However, EMR1 mRNA expression levels differed significantly between control LNs on one hand and both H&E(‐) (*p* < 0.0001) and H&E(+) (*p* = 0.02) LNs on the other.

To correlate EMR1 mRNA expression with patient survival, the LN with the highest EMR1 mRNA level was selected for each patient. Figure 2C,D displays the EMR1 mRNA expression profile for the 121 colon cancer patients and the 13 non‐cancerous controls. Only the differences for stage II LNs and H&E(‐) LNs remained.

Pairwise comparison revealed no significant correlation between EMR1 mRNA levels in primary colon cancer tumors and the corresponding highest LN (*r* = 0.02, *p* = 0.9), suggesting independent expression patterns.

### 2.3 Utility of Determining EMR1 mRNA Levels in Primary Tumor and in Lymph Nodes for Prediction of Recurrence After Surgery of Colon Cancer

**FIGURE 3:**
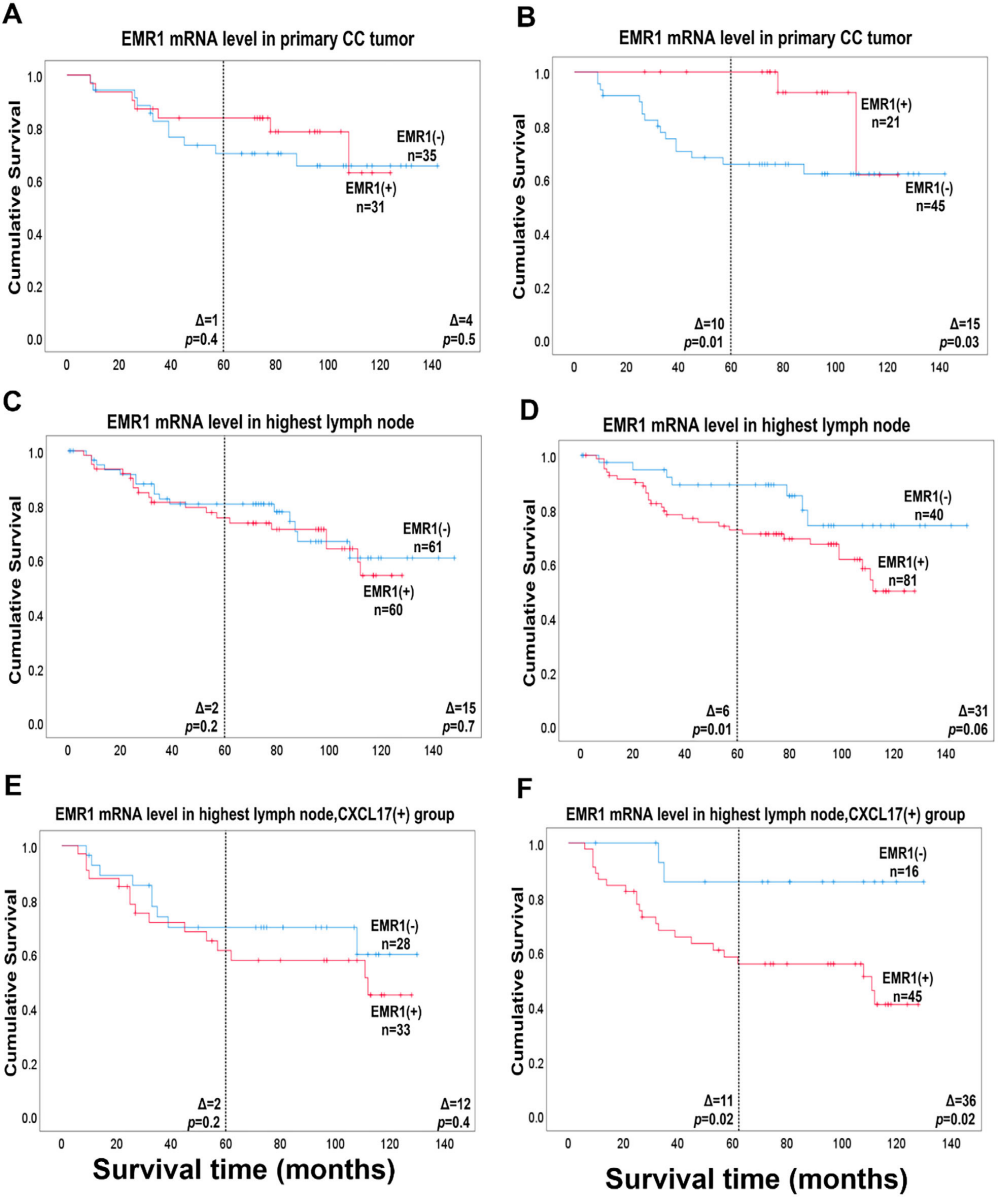
Cumulative recurrence curves according to Kaplan–Meier for colon cancer patients categorized to two groups according to EMR1 mRNA levels in primary tumor and lymph nodes. (A, B) patients categorized according to the EMR1 mRNA level in the primary tumor (*n* = 66). (C, D) all patients for whom the EMR1 mRNA levels were determined in lymph nodes (*n* = 121) categorized according to the EMR1 level in the highest lymph node. (E, F) patients with CXCL17 levels above 0.0003 mRNA copies per 18S rRNA unit in their lymph nodes (CXCL17(+) group; *n* = 61) categorized according to the EMR1 level in the highest lymph node. (A, C, E) median EMR1 level used as cut‐off. (B, D, F) percentile‐based adjusted cut‐off used. Red curves depict patients in the group with values above the respective cut‐off, EMR1(+). Blue curves depict patients in the group with values below the respective cut‐off, EMR1(‐). *n*‐values next to the curves show the number of patients in each group. The dashed vertical line indicates 5‐year follow‐up. Δ = difference in mean disease‐free survival time in months between the EMR1(+) and EMR1(‐) groups. Δ‐ and *p*‐values to the left in the graphs are from 5‐year follow‐up, and those to the right are from 12‐year follow‐up. *p*‐values were determined by log rank test, and a *p*‐value ≤ 0.05 was considered statistically significant.

The prognostic value of quantifying EMR1 mRNA levels in the primary tumor and in the highest LN of surgically treated colon cancer patients was investigated. We assessed differences in disease‐free survival time following surgery using the Kaplan–Meier survival model combined with the log‐rank test and determined the hazard risk ratio for recurrence using Cox regression analysis. To differentiate between patients expressing different mRNA levels of EMR1 two methods were used. A cut‐off level between EMR1‐positive, EMR1(+) and EMR1‐negative, EMR1(‐), patients was established either by 1) determining the median value for the entire group and 2) by establishing a percentile adapted cut‐off giving the best division between a high and low risk category by dividing patients into equally large subgroups based on their EMR1 mRNA level in the highest LN and comparing them pairwise with each‐other with regard to recurrency, combining those which are not significantly different from each‐other and so on, ending up with one subgroup different from all other combined. Figure 3A,C,E shows the results using the median value, and Figure 3B,D,F using the percentile adapted cut‐off. Figure 3A,B displays the results for EMR1 levels in the primary tumor. While the median value gave no difference between the high and low group (Figure 3A) the 68^th^ percentile (0.55 EMR1 mRNA copies/18S rRNA unit) gave a partition (Figure 3B). Mean survival time differed by 10 months between the high and low EMR1 group (*p* = 0.01) at 5‐year follow‐up. Note that high EMR1 levels were associated with good prognosis and that no patient in this group died in colon cancer before 6 years after surgery. The EMR1(+) group (*n* = 21) exhibited a 0.1‐fold reduction in recurrence rate at 5‐year follow‐up (*p* = 0.04) and 0.2‐fold at 12‐year follow‐up (*p* = 0.05) compared to the EMR1(‐) group (*n* = 45) (Table 1).

**TABLE 1:** Comparative analysis of average survival time after surgery and risk for recurrence of disease of all colon cancer patients in the study and colon cancer patients with high CXCL17 mRNA levels categorized according to EMR1 level in primary tumor and the highest lymph node.

Patient group	Category	Number of patients				Total	5‐year follow‐up after surgery					12‐year follow‐up after surgery				
		Stage I	Stage II	Stage III	Stage IV		Disease‐free survival^a^			Risk of recurrence^b^		Disease‐free survival^a^			Risk of recurrence^b^	
							Average (months)	Difference (months)	*p*‐value	Hazard ratio (95%CI)	*p*‐value	Average (months)	Difference (months)	*p*‐value	Hazard ratio (95%CI)	*p*‐value
**EMR1 mRNA level in primary colon cancer tumor**																
All CC^c^ patients	EMR1(‐)^d^	7	21	12	5	45	48	10	0.01	0.1	0.04	100	15	0.03	0.2	0.05
	EMR1(+)	7	9	5	0	21	58			(0.02–0.9)		115			(0.05–1.0)	
**EMR1 mRNA level in the highest lymph node**																
All CC patients	EMR1(‐)^e^	6	20	12	2	40	55	6	0.01	3.1	0.02	124	31	0.06	2.1	0.07
	EMR1(+)	17	32	25	7	81	49			(1.2–8.0)		93			(0.9–4.8)	
CXCL17(+) CC patients^f^	EMR1(‐)^e^	1	7	6	2	16	56	11	0.02	4.6	0.04	116	36	0.02	4.7	0.04
	EMR1(+)	8	16	15	6	45	45			(1.1–19.7)		80			(1.1–19.9)	

^a^ Mean survival time after surgery for colon cancer patients as calculated by cumulative survival analysis according to Kaplan–Meier.

^b^ Hazard ratio with 95% confidence interval (CI) for colon cancer patients as calculated according to univariate COX regression analysis.

^c^ CC = colon cancer.

^d^ Colon cancer patients divided into two groups EMR1(‐) and EMR1(+) according to the 68^th^ percentile of mRNA levels of primary tumors (0.5497 mRNA copies/18S rRNA unit).

^e^ Colon cancer patients divided into two groups EMR1(‐) and EMR1(+) according to the 33^rd^ percentile of mRNA levels in the highest lymph node (0.2864 mRNA copies/18S rRNA unit).

^f^ CXCL17(+) group, colon cancer patient group with CXCL17 mRNA levels above 0.0003 mRNA copies/18S rRNA unit.

Contrary to the findings for primary tumors, in LNs high EMR1 mRNA levels were associated with an unfavorable prognosis. Patients stratified into EMR1(+) and EMR1(‐) groups based on the 33^rd^ percentile (0.29 EMR1 mRNA copies/18S rRNA unit) exhibited a significantly reduced mean survival time of 6 months at 5‐year follow‐up (*p* = 0.01) (Figure 3D). The EMR1(+) group (*n* = 81) exhibited a 3.1‐fold increase in recurrence rate at 5‐year follow‐up (*p* = 0.02) compared to the EMR1(‐) group (*n* = 40) (Table 1). Using the median value as a cut‐off achieved no division (Figure 3C).

### 2.4 Correlations Between the EMR1 mRNA Expression Level and CEA, CXCL16, CXCL17, POSTN, and LGR6 mRNA Expression Levels in Primary Tumors and Lymph Nodes of Colon Cancer Patients

**FIGURE 4:**
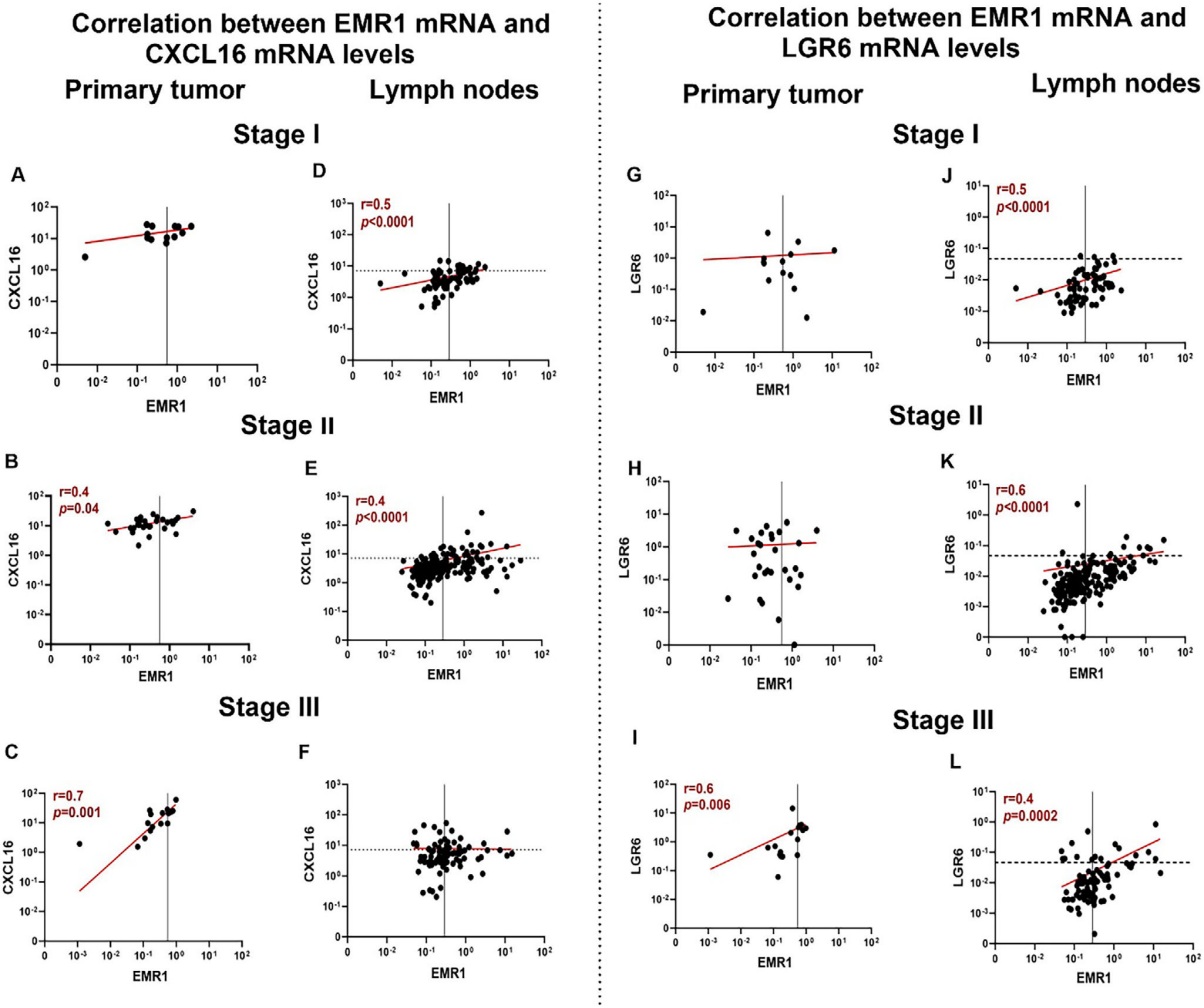
Correlation between EMR1 mRNA levels and CXCL16 mRNA levels (A–F) and LGR6 mRNA levels (G–L) in primary tumor of 61 colon cancer patients, 14 in TNM stage I (A, G), 30 in stage II (B, H), and 17 in stage III (C, I) and in the highest lymph node of 112 colon cancer patients, 23 in stage I (D, J), 52 in stage II (E, K) and 37 in stage III (F, L). Each dot represents one tumor and one lymph node, respectively. mRNA levels are given as mRNA copies per 18S rRNA unit. (r) and *p*‐values from two‐sided Spearman rank correlation test are given for correlations with *p*‐values ≤ 0.05. Vertical lines in (A–C) and (G–I) depict the 68^th^ percentile of EMR1 mRNA in primary tumor tissue, and in (D–F) and (J–L) depict the 33^rd^ percentile of EMR1 mRNA in lymph nodes, i.e., the adapted cut‐offs for allocation of patients to EMR1(+) and EMR1(‐) groups in survival analysis. Hatched horizontal lines in (D–F) and (J–L) depict cut‐off for CXCL16 and LGR6, respectively, in prognostic analysis using lymph nodes.

The mRNA expression levels of the biomarkers listed above have previously been determined in the same clinical material of primary tumors and LNs studied here [21, 23, 24, 25, 26]. Correlation coefficients for all comparisons between EMR1 expression levels in primary tumors and LNs of different TNM stages and CEA, CXCL16, CXCL17, POSTN, and LGR6 mRNAs in corresponding tissues, respectively, are shown in Table 2. Detailed data for the correlation between EMR1 on one hand and CXCL16 and LGR6 on the other are shown in Figure 4. The results can be summarized as follows: (1) mRNA expression levels of EMR1 did not correlate with the levels of CEA (CEACAM5), with one exception, namely LNs of stage I patients. (2) The chemokines CXCL16 and CXCL17 both correlated with EMR1 in both primary tumors and LNs, with the strongest correlation to CXCL16. In LNs, this correlation was observed for stage I and II patients but not for stage III patients. For CXCL16, this contrasted with the finding in primary tumors, where stage III patients showed a strong correlation with EMR1. (3) For POSTN there was no correlation with EMR1 in LNs but a correlation in stage II and III primary tumors. (4) LGR6 was strongly correlated to EMR1 in LNs in stages I–III and to primary tumors in stage III.

**TABLE 2:** Correlations between levels of EMR1 mRNA and CEA, CXCL16, CXCL17, POSTN, and LGR6 mRNA levels in the primary tumor and lymph nodes of colon cancer patients.

	Tissue type and CC patient group	CEA		CXCL16		CXCL17		POSTN		LGR6	
		r	*p*	r	*p*	r	*p*	r	*p*	r	*p*
EMR1	Primary tumor All (*n* = 66)	0.3	NS	0.5	<0.0001	0.2	0.05	0.5	0.0006	0.1	NS
	Primary tumor Stage I (*n* = 14)	0.5	NS	0.3	NS	0.2	NS	0.1	NS	0.1	NS
	Primary tumor Stage II (*n* = 30)	0.1	NS	0.4	0.04	0.2	NS	0.7	0.002	0.0	NS
	Primary tumor Stage III (*n* = 17)	0.6	NS	0.7	0.001	0.5	NS	0.6	0.05	0.6	0.006
	Lymph node All (*n* = 377)	0.0	NS	0.4	<0.0001	0.2	0.005	−0.0001	NS	0.5	<0.0001
	Lymph node Stage I (*n* = 70)	0.3	0.004	0.5	<0.0001	0.4	0.0006	−0.02	NS	0.5	<0.0001
	Lymph node Stage II (*n* = 189)	−0.1	NS	0.4	<0.0001	0.2	0.005	−0.04	NS	0.6	<0.0001
	Lymph node Stage III (*n* = 88)	0.1	NS	0.1	NS	−0.1	NS	0.1	NS	0.4	0.0002

The correlation coefficients (r) and the *p*‐values (*p*) were calculated by two‐tailed Spearman's rank order correlation test using levels given as mRNA copies/18S rRNA unit. CC = colon cancer. *n* = number of patients. NS = Not statistically significant, *p*‐value > 0.05.

### 2.5 Combining Biomarkers for Prediction of Recurrence

To investigate if EMR1 mRNA analysis of LNs could be used to achieve further division of patients, who have already been identified as a group with poor prognosis, we subjected patients with CXCL17 mRNA levels above the cut‐off, that is > 0.0003 CXCL17 mRNA copies/18S rRNA unit [21], to EMR1 mRNA analyses. Using the median EMR1 mRNA value as cut‐off, no significant difference between the high and low group was seen (Figure 3E). However, using the 33^rd^ percentile as cut‐off, a highly significant difference between the high and low EMR1 mRNA expression group was seen (11 months at 5‐year follow‐up; *p* = 0.02) (Figure 3F). Cox regression analysis revealed a 4.6‐fold (5‐year follow‐up) higher recurrence rate in EMR1(+) patients (*n* = 45) compared to EMR1(‐) patients (*n* = 16) (*p* = 0.04; Table 1). Thus, by using this double biomarker procedure, a subgroup with a very unfavorable prognosis could be identified.

### 2.6 Demographic‐, Clinical‐ and Prognostic Factors and EMR1 mRNA Levels in Either the Primary Tumor or the Highest Lymph Node as Risk Indicators for Recurrence

Risk analysis was performed by Cox regression uni‐ and multivariate analysis for site of lesion, primary tumor grade, H&E status of LNs, TNM stage, age, sex, and postoperative adjuvant treatment in two patient cohorts, one for EMR1 mRNA levels in primary tumor (*n* = 66) and one for EMR1 levels in highest LNs (*n* = 121). In primary tumors, high EMR1 mRNA expression levels were associated with improved overall survival in the univariate analysis at both 5 and 12‐year follow‐up. In addition, positive H&E status, TNM stage I/II versus III/IV, and no postoperative adjuvant treatment were associated with poor prognosis at both timepoints of follow‐up. However, all these parameters lost their statistical significance in multivariate analysis, although a good prognosis for high EMR1 levels was close at 5‐years (*p* = 0.06) (Table 3).

**TABLE 3:** Comparison between demographic‐, clinical‐ and prognostic factors and EMR1 mRNA levels in either primary tumor or the highest lymph node as risk indicators for recurrence of colon cancer.

Variable	Uni‐variate analysis (n^a^)	Multi‐variate analysis (n)	5‐year follow‐up after surgery				12‐year follow‐up after surgery			
			Univariate analysis		Multivariate analysis		Univariate analysis		Multivariate analysis	
			Hazard ratio (95% CI^b^)	*p*‐value	Hazard ratio (95% CI)	*p*‐value	Hazard ratio (95% CI)	*p*‐value	Hazard ratio (95% CI)	*p*‐value
		**EMR1 mRNA level in the primary tumor**								
**EMR1 mRNA level category (high vs. low)**	21/45	21/44	0.1 (0.02–0.9)	0.04	0.1 (0.02–1.1)	0.06	0.2 (0.05–1.0)	0.05	0.3 (0.06–1.3)	0.1
**Sex (male vs. female)**	30/36	29/36	1.4 (0.5–3.5)	0.5	0.9 (0.3–2.4)	0.8	1.4 (0.6–3.6)	0.5	0.9 (0.3–2.6)	0.8
**Age (*<*74 vs. ≥74 years)** ^c^	39/27	39/26	0.9 (0.4–2.6)	0.9	1.7 (0.3–9.4)	0.5	1.0 (0.4–2.7)	0.9	1.5 (0.3–6.6)	0.6
**Site of lesion (right vs. left)**	38/28	37/28	0.7 (0.3–1.8)	0.4	0.6 (0.2–1.8)	0.3	0.6 (0.2–1.6)	0.3	0.6 (0.2–19.3)	0.3
**Primary tumor grade (high vs. low)**	53/12	53/12	0.5 (0.2–1.3)	0.1	0.6 (0.2–2.0)	0.4	0.5 (0.2–1.2)	0.1	0.7 (0.2–2)	0.5
**H&E status (positive vs. negative)**	10/56	10/55	4.0 (1.5–10.9)	0.006	2.3 (0.6–8.7)	0.2	4.1 (1.5–11.0)	0.005	2.5 (0.6–10.0)	0.2
**TNM stage (I/II vs. III/IV)**	44/22	43/22	3.5 (1.3–9.1)	0.01	1.3 (0.3–6.2)	0.7	3.1 (1.2–7.8)	0.02	0.9 (0.2–4.5)	0.9
**Postoperative adjuvant treatment (no vs. yes)**	52/14	51/14	3.8 (1.5–9.6)	0.005	3.6 (0.6–22.1)	0.2	4.1 (1.5–11.0)	0.005	3.5 (0.6–19.3)	0.2
		**EMR1 mRNA level in the highest lymph node**								
**EMR1 mRNA level category (high vs. low)**	81/40	81/38	3.1 (1.2–8.0)	0.02	4.2 (1.5–11.7)	0.006	2.1 (0.9–4.8)	0.07	3.3 (1.3–8.2)	0.01
**Sex (male vs. female)**	55/66	54/65	1.4 (0.7–2.8)	0.3	1.4 (0.7–3.0)	0.4	1.6 (0.8–3.1)	0.2	1.6 (0.8–3.5)	0.2
**Age (*<*74 vs. ≥74 years)** ^c^	73/48	73/46	0.7 (0.4–1.5)	0.4	1.3 (0.4–4.1)	0.6	0.9 (0.5–2.0)	0.9	1.6 (0.6–4.5)	0.3
**Site of lesion (right vs. left)**	71/50	69/50	0.7 (0.4–1.5)	0.4	1.3 (0.6–2.9)	0.5	0.7 (0.4–1.4)	0.4	1.3 (0.6–2.7)	0.6
**Primary tumor grade (high vs. low)**	97/22	97/22	0.2 (0.09–0.4)	<0.001	0.2 (0.1–0.5)	<0.001	0.3 (0.1–0.5)	<0.001	0.3 (0.1–0.6)	0.002
**H&E status (positive vs. negative)**	18/103	18/101	4.2 (2.0–8.7)	<0.001	2.1 (0.9–5.0)	0.09	3.5 (1.7–7.1)	<0.001	1.9 (0.8–4.7)	0.2
**TNM stage (I/II vs. III/IV)**	75/46	73/46	5.9 (2.8–12.8)	<0.001	3.9 (1.3–11.7)	0.02	4.9 (2.5–10.0)	<0.001	3.4 (1.2–9.8)	0.02
**Postoperative adjuvant treatment (no vs. yes)**	94/27	92/27	4.4 (2.2–8.8)	<0.001	0.9 (0.3–3.6)	0.9	3.8 (2.0–7.4)	<0.001	1.1 (0.3–3.7)	0.8

^a^ *n* = number of patients in the indicated category.

^b^ CI = confidence interval.

^c^ The median age, 74 years, was used as cut‐off.

In contrast, when multivariate analysis was performed on the high and low EMR1 expression groups of the highest LN, the risk for recurrence increased (hazard ratio 3.1 in univariate‐ and 4.2 in multivariate analysis; Table 3) and statistical significance became even stronger (*p* = 0.006 at 5 years; Table 3). Additionally, primary tumor grade had a significant prognostic value, which remained in multivariate analysis, suggesting that it is an independent prognostic factor relative to EMR1 (Table 3). TNM stage was significant in univariate analysis but decreased in both hazard risk ratio and *p*‐value in multivariate analysis, suggesting partial overlap with EMR1 (Table 3).

## 3 Discussion

We found that the prognostic significance of EMR1 mRNA expression levels depends on whether it is the primary tumor or the LN that is analyzed. In contrast to EMR1 analysis of the primary tumor, it appears that EMR1 analysis of LNs has prognostic value. Thus, a high EMR1 level is a strong, independent indicator of risk for recurrence within 5 years after curative surgery of colon cancer, underscored by increased hazard ratio and statistical significance in multivariate‐ compared to univariate analysis. The difference between the EMR1‐high and EMR1‐low groups was even more pronounced in the subgroup of patients exhibiting high levels of both EMR1 and CXCL17 mRNA, another biomarker that is also an indicator of poor prognosis [21]. This finding demonstrates that consecutive biomarker analysis allows identification of patients with a very poor prognosis. The size of the latter group was 37% in this clinical material. Patients were evenly distributed among stage I–III patients, and almost all stage IV patients were found in this group. Probably, the inclusion of additional biomarkers of poor prognosis could give a more accurate identification of extremely high‐risk patients who would need immediate clinical attention.

A rare finding for EMR1 was that mRNA levels in the primary tumor appeared to have a value for assessment of outcome, something we have not seen with previously studied biomarkers. Thus, several biomarkers with prognostic value when determined in LNs proved to have no or very low prognostic value when assessed in the primary tumor [21, 22, 24, 25, 26, 27], possibly suggesting a unique situation for EMR1. However, the results from multivariate analysis, in which EMR1 high vs. low levels were compared with seven clinicopathological and demographic parameters, indicated a slight loss of significance—from a *p*‐value of 0.04 to 0.06 at 5 years follow‐up. Notably, high EMR1 mRNA levels in primary tumors were a sign of good prognosis. While it cannot be excluded that these findings can be explained by the relatively small group of primary tumors from colon cancer patients (*n* = 66) that was analyzed, other explanations for the result are also possible. EMR1 may be expressed in partly different cell types at the two sites, an assumption that is supported by the finding that there was no correlation between EMR1 levels in the primary tumor and LNs. Previous studies by Prizment and coworkers have shown that eosinophil infiltration in CRC primary tumors predicts favorable outcomes [10], which is in line with this study. Since EMR1 is produced by eosinophils, it is likely that the high EMR1 levels in primary tumors reflect eosinophil infiltration in addition to ectopic expression in tumor cells. Interestingly, metastatic LNs of CRC patients were reported to harbor no or very few eosinophils [14]. Thus, the anti‐tumor effects exerted by eosinophils might be lacking in the LNs, while the 10%–15% tumor cells that ectopically express EMR1 in the LNs may exert effects that promote tumor cell survival [15]. An important issue is to what extent ectopic expression influences the functions of the tumor cell, for instance, if tumor cells expressing EMR1 gain features of myeloid cells that promote LN metastasis. EMR1 mRNA expression demonstrated a strong correlation with the myeloid‐derived chemokines CXCL16 and CXCL17, supporting this notion.

Ectopic expression of biomarkers is not as simple a concept as it may seem. Even if one can be sure that the biomarker is produced in the tumor cells and not in mature normal cells of the same lineage—here, normal colonocytes—it may not be possible to ascertain that immature cells of the same lineage do not express the biomarker at low or very low levels, thus precluding differentiation between true ectopic expression and expression in immature cells. The latter possibility might apply to EMR1, which is very strongly correlated with LGR6, a stem cell marker, and is expressed in LNs, while there was a poor correlation in the primary tumor, where the main source of EMR1 mRNA is most likely eosinophils.

The EMR1 mRNA levels were lower in LNs from colon cancer patients compared to LNs from controls. One possible explanation for this result is that all but one control patient had inflammatory bowel disease, and the inflammatory state of these patients often also affects the regional LNs. This means that there could be a spread of myeloid cells from the gut mucosa to the LNs in controls. Not only eosinophils but also monocytes/macrophages and basophils may be the source of EMR1 in such LNs since these cell types also express EMR1, although at lower levels than eosinophils [16].

The present study illustrates the usefulness of using a percentile‐based cut‐off for allocation of patients to high‐ and low‐risk groups in survival analyses. The precise value of the cut‐off is dependent on the size of the clinical material. Thus, additional studies in independent, larger clinical materials are needed before a final cut‐off can be established.

## 4 Conclusion

EMR1 expression in primary colon cancer tumors is probably mainly due to infiltrating eosinophils, although it is also ectopically expressed in the tumor cells. In LNs, however, where eosinophils are essentially lacking, EMR1 expression is dominated by the tumor cells. This might reflect two parallel ongoing processes, one anti‐tumor action taking place in the primary tumor depending on the infiltrating eosinophils and another taking place in both regional LNs and the primary tumor, where EMR1 might act as a receiver of external signals promoting tumor growth, for instance by allowing uptake of Ca^2+^ ions. At any rate, EMR1 mRNA analyses can serve as a useful prognostic marker for poor outcome in LNs, particularly if combined with another marker for poor outcome.

## 5 Material and Method

### 5.1 Patient Population and Tissue Samples for mRNA Expression Profiling

LN samples (*n* = 377) were obtained from 121 colon cancer patients representing different TNM stages. According to histopathology using hematoxylin and eosin staining (H&E), 357 LNs were non‐metastatic [H&E (‐)], and 20 LNs were metastatic [H&E (+)]. Preoperative treatment was not administered to any of the patients. Control patients (*n* = 13) provided 77 control LNs. Control patients had underlying conditions: Crohn's disease (*n* = 1), lipoma (*n* = 1), and ulcerative colitis (*n* = 11). Fresh primary colon cancer tumors were available from sixty‐six of these patients. Normal colon tissue samples were harvested from the primary colon cancer tumor resection margins (*n* = 30). Tissues were frozen post‐surgical resection promptly and maintained at −70°C until RNA extraction. Table 4 summarizes the demographic and clinicopathological features of patients contributing tissue samples for mRNA expression profiling.

**TABLE 4:** Demographic and clinicopathological features of colon cancer (CC) and control patients contributing tissue samples for mRNA expression profiling.

Demographic and clinicopathological features		Primary CC tumor tissue	Normal colon tissue	Lymph nodes of CC patients^a^	Lymph nodes of control patients^b^
N^c^		66	30	121	13
Sex	Male	30	17	55	10
	Female	36	13	66	3
Age	Median	74	72	74	23
	Min‐max	42‐89	57‐85	42‐89	9‐32
TNM	I	14	7^d^	23	– ^e^
	II	30	17	52	—
	III	17	4	37	—
	IV	5	2	9	—

^a^ The number of lymph nodes retrieved was 377. Of these, 70 lymph nodes came from 23 patients in stage I, 189 lymph nodes from 52 patients in stage II, 88 lymph nodes from 37 patients in stage III, and 30 lymph nodes from 9 patients in stage IV.

^b^ The number of lymph nodes retrieved was 77.

^c^ N = number of patients who had contributed tissue samples of primary colon cancer tumor tissue, normal colon tissue, and lymph nodes of colon cancer and control patients, respectively.

^d^ Normal colon tissue was from the resection margins of colon cancer tumors of the indicated TNM stages.

^e^ – = not applicable.

### 5.2 Patient Population and Tissue Samples for Immunohistochemistry

Ten colon cancer fresh tumor samples (5 women and 5 men with a median age of 72 years, ranging from 60 to 84 years) were used for immunohistochemistry. Patient tumor stages: Stage I (1 patient), Stage II (3 patients), Stage III (4 patients), and Stage IV (2 patients). The samples underwent phosphate‐buffered saline washing at 4°C, snap‐freezing in iso‐pentane precooled in liquid nitrogen, and were thereafter stored at ‐70°C for section preparation.

### 5.3 Real‐Time qRT‐PCR Analysis

To quantify EMR1 mRNA concentrations, we established a real‐time qRT‐PCR assay with a specific RNA copy standard, specific primers in distinct exons, and a reporter dye‐labeled probe that hybridized over the exon border in the amplicon. The EMR1 mRNA assay recognizes all five known transcript variants (NM_001974, NM_001256252, NM_001256253, NM_001256254, NM_001256255). The primer and probe sequences were forward primer 5ˊ‐ATAAGACCCACACGGAAACCA‐3ˊ, reverse primer 5ˊ‐AAGCTGGGCACAAGGTACTG‐3ˊ, and probe 5ˊ‐CACAAAGGGTAATAAC‐3ˊ. MGB served as the quencher dye, and FAM as the reporter dye. The amplicon measured 67 bases. The qRT‐PCR reaction was done at 60°C for 5 min and 95°C for 1 min, followed by 45 cycles of 95°C for 15 s and 60°C for 1 min. The RNA copy standard was a synthetic RNA oligonucleotide (Dharmacon, Lafayette, CO, USA) with the same sequence as the amplified region in the qRT‐PCR assay. Real‐time qRT‐PCR assays for CEA, CXCL17, CXCL16, POSTN, and LGR6 mRNAs were previously described [21, 23, 24, 25, 26, 27]. The specific RNA copy standard was serially diluted at concentrations ranging from 10^3^ to 10^8^ copies/µL for every qRT‐PCR run. The standard curve was used to determine the concentrations in unknown samples, which were then expressed as copies of mRNA/µL. The concentration of 18S rRNA was measured in each sample using real‐time qRT‐PCR (Applied Biosystems, Foster City, CA, USA) to normalize mRNA levels as described [28]. Quantification of 18S rRNA was achieved using a standard curve generated from serially diluted total RNA extracted from human peripheral blood mononuclear cells. One unit was defined as the amount of 18S rRNA in 10 pg RNA [28]. Expression levels were given as mRNA copies/18S rRNA unit.

### 5.4 Antibodies and Substrate

Immunohistochemistry utilized rabbit anti‐EMR1 serum (NBP231090, Novus, Littleton, CO, USA) as the primary antibody and ImmPress anti‐rabbit IgG HRP‐conjugate (MP‐7401, Vector Laboratories) as the secondary reagent, with 3,3′‐diaminobenzidine (DAB, Vector Laboratories) as the peroxidase substrate.

### 5.5 Immunohistochemistry

Frozen tissues were sectioned (4–6 µm) and processed as previously reported [20, 29]. Briefly, tissue sections underwent fixation (4% paraformaldehyde), blockage of endogenous peroxidase activity (0.03% hydrogen peroxide, 2 mm sodium azide), blocking of non‐specific binding sites (0.2% BSA, 2.5% horse serum), and sequential incubation with primary antibody, ImmPress anti‐rabbit IgG, and DAB (0.05%) as substrate. Finally, the sections are counterstained by methyl green.

### 5.6 Statistical Analysis

qRT‐PCR results were normalized by dividing the concentration of RNA copies of the mRNA species under investigation with the concentration of the house‐keeping gene 18S rRNA in the same sample, thereby giving the level of the mRNA as copies/18S rRNA unit. No other pre‐processing was performed. We used non‐parametric statistical tests because most sample sets did not have a Gaussian distribution as estimated by the Shapiro‐Wilk test. Descriptive values are given as median and min–max. Outliers were retained in the analyses.

The Mann–Whitney U test was used for comparisons between two groups, including comparisons of EMR1 mRNA levels between normal colon tissues (*n* = 30) and primary colon cancer tumors (*n* = 66), as well as comparisons of the highest EMR1:CEA ratio between [H&E(+), *n* = 13] and [H&E(‐), *n* = 108] LNs. For multiple group comparisons, the Kruskal–Wallis test was performed followed by Dunn's post hoc multiple comparison test with adjustment for multiple testing. This included analyses of EMR1 mRNA levels in control LNs (*n* = 77) and LNs from different patient groups (totally 377 LNs). Correlations between EMR1 mRNA levels and CEA, CXCL17, CXCL16, POSTN and LGR6 mRNA levels in primary tumors and LNs, as well as correlations between EMR1 expression in primary tumors and corresponding highest LNs, were assessed using Spearman's rank correlation coefficient test. The GraphPad Prism 9 software (GraphPad Software, San Diego, CA, USA) was used for comparative analyses of mRNA levels.

Survival analyses were performed comparing patient groups for mean disease‐free survival time after surgery according to the Kaplan–Meier cumulative survival model in combination with the log‐rank test. Risk for recurrent disease after surgery was estimated as hazard ratio with 95% confidence interval (CI) using univariate and multivariate Cox regression analysis. A panel of clinical variables plus age and sex were analyzed. Cases with missing values in multivariate analysis were 1.5% for the group with EMR1 levels in the primary tumor and 1.6% in the group with EMR1 levels in LNs. Patients who died from causes other than colon cancer were considered disease‐free. The SPSS software (IBM Corporation, Armonk, NY, USA) was used for survival analyses.

Two‐sided analyses were used throughout, and a *p*‐value of ≤ 0.05 was considered statistically significant.

### 5.7 Ethical Considerations

All techniques used in studies involving human participants agreed with the ethical standards of the institutional research committee and with the 1964 Helsinki Declaration and its later amendments and comparable ethical standards. Tumor samples and LNs were collected after patients’ written informed consent. This study was authorized by the Local Ethics Research Committee of the Medical Faculty, Umeå University, Umeå, Sweden. Approved dates and registration numbers: December 3, 2003 (03‐503) and May 3, 2023 (2023‐01396‐01).

## Author Contributions

**Tamer Roshdy**: data curation and Writing – original draft. **Hagar Eltorky**: data curation, writing, review, and editing. **Alaa Mohamed**: statistical analysis and writing, review and editing. **Faten Zahran**: supervision, writing, review, and editing. **Adel Guirgis**: supervision, writing, review, and editing. **Lina Olsson**: data curation, writing, editing, and review. **Gudrun Lindmark**: writing – review, editing, and data curation. **Marie‐Louise Hammarström**: writing – original draft, review, editing, data curation, conceptualization, and resources. **Sten Hammarström**: conceptualization, data curation, writing – original draft, review, editing, and visualization. **Basel Sitohy**: supervision, conceptualization, writing, review, and editing, visualization, resources, data curation, and project administration .

## Funding

The author(s) state that financial assistance was provided for the authoring, research, and publication of the current study. Grants for this study were provided by the Medical Faculty of Umeå University (M‐LH and BS), Kempe Foundation (BS), Grant number JCK22‐0003, the County Council of Västerbotten (BS) RV‐995803, the Swedish Research Council‐Medicine and Health (BS) Grant number 2008–7042, the Lions Cancer Research Fund (BS), Grant number LP 24–2373, the Swedish Research Council‐Natural and Engineering Sciences (M‐LH) Grant numbers 2013‐04522 and 2010–05669, and the Stig and Ragna Gorthon Foundation (GL).

## Conflicts of Interest

The authors declare no conflicts of interest.

## Data Availability

The data that support the findings of this study are available from the corresponding author upon reasonable request.
